# Editorial: Microbial biosurfactants: updates on their biosynthesis, production and applications

**DOI:** 10.3389/fbioe.2024.1433035

**Published:** 2024-06-05

**Authors:** Rudolf Hausmann, Eric Déziel, Gloria Soberón-Chávez

**Affiliations:** ^1^ Department of Bioprocess Engineering, Institute of Food Science and Biotechnology, University of Hohenheim, Stuttgart, Germany; ^2^ Centre Armand-Frappier Santé Biotechnologie, Institut National de la Recherche Scientifique, Université du Québec, Laval, QC, Canada; ^3^ Instituto de Investigaciones Biomédicas, Universidad Nacional Autónoma de México, Mexico City, Mexico

**Keywords:** biosurfactants, glycolipid, lipopeptides, renewable resources, sustainable development

Microbial biosurfactants are increasingly attracting interest as ingredients in green household and personal care products as well as in industrial and environmental biotechnological applications. This is due to their general surfactant efficacy and additional application-specific benefits, which have been demonstrated in numerous studies for available biosurfactants. The general advantages of biosurfactants include very good biodegradability, low toxicity, production from renewable raw materials and the environmental benefits perceived by consumers.

Traditional and widely used natural surfactants include lecithins, saponins (e.g., from the soap nut, *Sapindus* sp.) or surfactants derived from renewable raw materials such as soap (vegetable or animal fatty acid salts), betaine surfactants and alkyl polyglycosides (APGs). In contrast, microbial biosurfactants are derived from fermentation based on renewable raw materials. Currently, however, there is still a limited choice of widely available microbial biosurfactants, mostly represented by three glycolipids: sophorolipids, rhamnolipids and mannosylerythritol lipids. As the demand for fully biodegradable ingredients from renewable plant sources will certainly continue to increase, especially regarding the replacement of sulfate-based surfactants, sustainable alternatives that respond to consumer ethical concerns are needed; microbial biosurfactants precisely meet these requirements. For these reasons, research and development into biosurfactants is particularly important at the moment. Correspondingly the utilization of renewable substrates and challenges of sustainability are the common thread running through this Research Topic, which presents selected state-of-the-art contributions in the area of biosurfactants. It is linked to the second Biosurfactants International Conference, which took place from September 28th - 30th, 2022 in Stuttgart Hohenheim, Germany.

This Research Topic deals specifically with the characterization, analysis, biosynthesis, purification and life cycle assessment of glycolipid and lipopeptide biosurfactants. A first thematic focus of the 15 Research Topic articles is represented by the glycolipids. Nakamichi et al. provide structural insight into the catalytic domain of an acyltransferase involved in MEL biosynthesis by the basidiomycetous yeast *Pseudozyma tsukubaensis*. Bippus et al. evaluated the environmental impact of MEL production through a life cycle assessment (LCA) analysis and reported that the substrates, energy requirements for bioreactor aeration and solvents used for purification are the main contributors to the environmental impact. Eras-Muñoz et al. report on the use of industrial wastes as alternative feedstocks for the sustainable production of sophorolipids via solid-state fermentation. Most microbial biosurfactants naturally display a specific but limited structural variability, which restricts their properties and areas of application. Pala et al. have addressed this challenge by chemically modifying microbially produced sophorolipids to produce a series of amines and hydrogenated derivatives, then testing their antimicrobial properties. Glycine glycolipid, a little-studied biosurfactant from *Alcanivorax borkumensis*, has been studied by Karmainski et al. and they report on achieving improved growth rates and production kinetics. Another little-studied glycolipid class was addressed by Haala et al. who developed and optimized a minimal medium for the production of liamocins, polyol lipids biosurfactant produced by the yeast-like fungus *Aureobasidium pullulans*. Kumar et al. report on their study on a novel *Starmerella* species capable to produce sophorolipids, also able to reduce naphthyl ketones to their corresponding alcohols.

Lipopeptides represent the second research focus of this Research Topic. Treinen et al. demonstrate the applicability of an external foam column for *in situ* product removal of surfactin during fermentation of *Bacillus subtilis*. Moldes et al. report on the purification of *Bacillus* lipopeptides from complex matrices using polyacrylamide gel electrophoresis. Bochynek et al. analyzed the formation and structural features of surfactin micelles, highlighting the relationship between the structure and properties of various congeners. The bioactivity potential of *Bacillus* and *Pseudomonas* and their related lipopeptides is addressed in three contributions. Mukadam et al. report on a strain of *B. proteolyticus* and its biosurfactant, a blend of glycolipids and lipopeptides, that controls the growth of phytopathogen fungus *Sclerotium rolfsii* while Akintayo et al. report on the antifungal properties of two new lipopeptide-producing *B*. *velezensis* strains. Zhou et al. review the structure and function of *Pseudomonas* lipopeptides displaying surfactant and antimicrobial properties and examine how their biosynthesis could be controlled through a better understanding of regulation.

Finally, studies of general interest for biosurfactant research are also presented in a couple of articles: Jimoh et al. review the use of biosurfactants as anti-biofilm agents in industrial water systems through their biocidal and dispersant properties, and as anti-fouling and anti-corrosion agents, while Sass et al. present a simple method for quantification of anionic biosurfactants in aqueous solutions.

Overall, we are very pleased by the quality, diversity, and originality of scientific contributions compiled in this Research Topic. We believe it provides a representative cross-section of current research topics on microbial biosurfactants.

